# P-1396. A Decade of Tuberculosis in Cancer Patients: Trends from Panama’s National Oncology Institute

**DOI:** 10.1093/ofid/ofaf695.1583

**Published:** 2026-01-11

**Authors:** Monica R A Pachar Flores, Ricardo Gollini, Anabelle Drake, María Alejandra Rengifo, Gissel A Figueroa, Gabriella E Papineau, Diana I Tejera

**Affiliations:** Hospital Santo Tomas, Instituto Oncologico Nacional, Panama, Panama, Panama; Associate Investigator, Centro de Vacunación e Investigación (CEVAXIN), Panama City, Panama, Panama; Hospital Irma de Lourdes Tzanetatos, Panama City, Panama, Panama; Medical Intern, Complejo Hospitalario Dr. Arnulfo Arias Madrid, Panama, Panama, Panama; Hospital Irma de Lourdes Tzanetatos, Panama City, Panama, Panama; Irma De Lourdes Tzanetatos Hospital, Panama, Panama, Panama; Complejo Hospitalario Dr. Arnulfo Arias Madrid, Panama, Panama, Panama

## Abstract

**Background:**

TB remains a public health concern in Panama (47 cases/100,000). Cancer patients are at higher risk due to immunosuppression from HIV, chemotherapy, and corticosteroids. Despite 155,917 consultations and 50,306 radiotherapy treatments at ION in 2022, TB prevalence in oncology patients is understudied. This study examines TB’s clinical and epidemiological profile in ION cancer patients (2013-2022).

**Figure:**
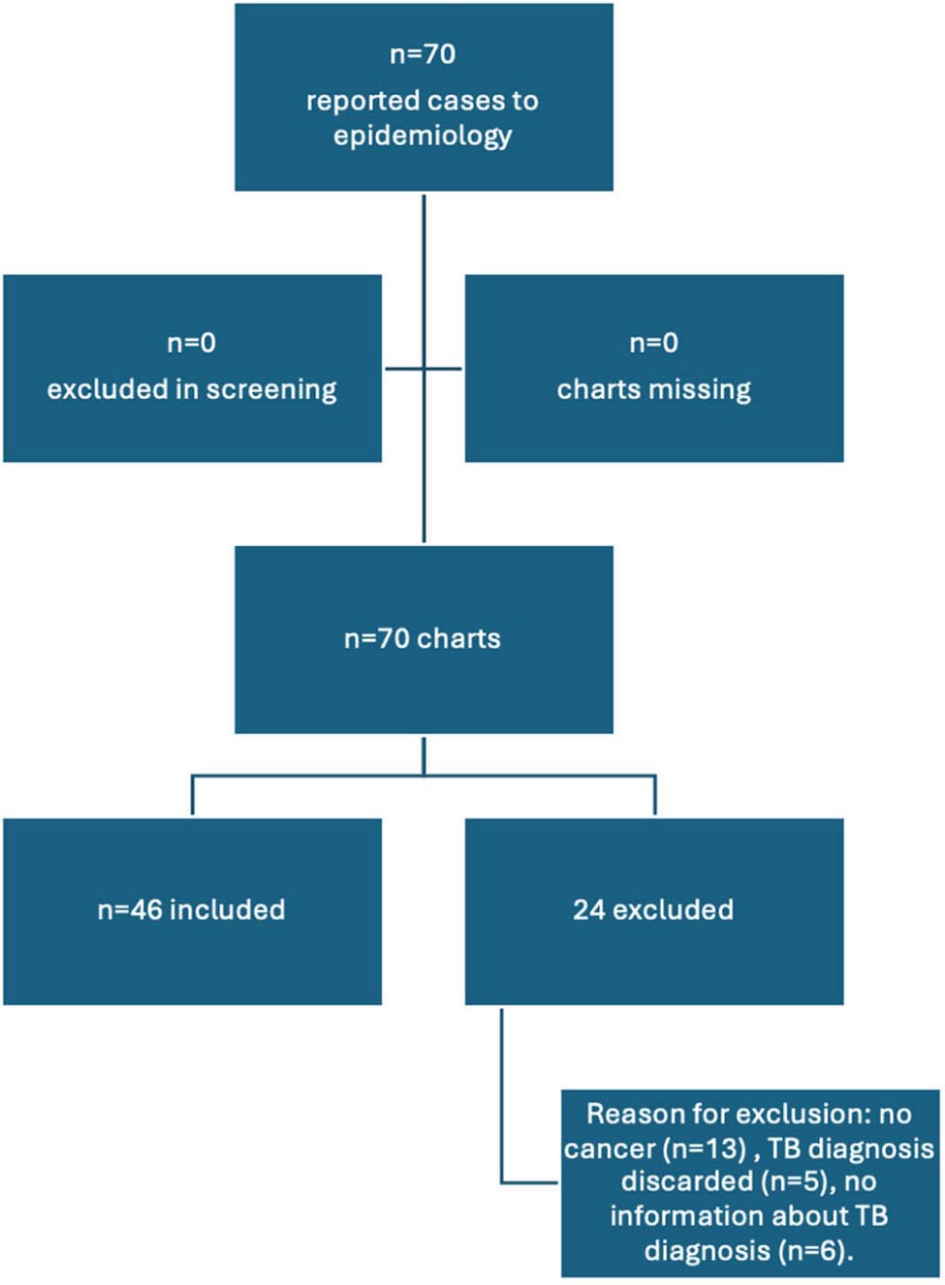
Eligibility and Selection of Tuberculosis Cases Reported at the Instituto Oncologico Nacional from 2012-2022

**Figure:**
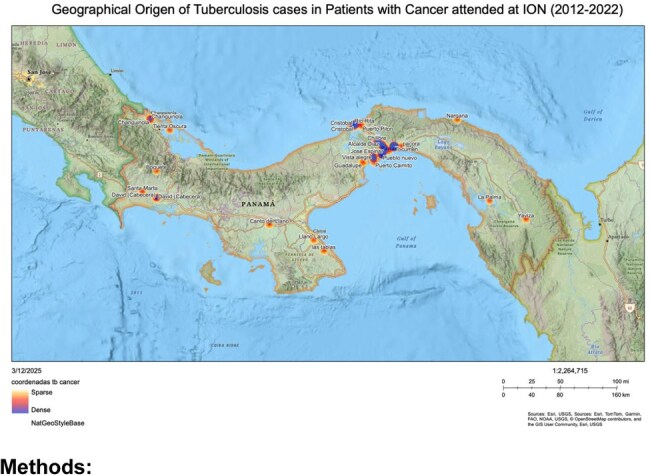

**Methods:**

A retrospective study analyzed TB cases in cancer patients (2013-2022) using ION’s notifiable disease database. Data were extracted from electronic health records and analyzed with Excel, Epi Info 7.2.5, and Python. Descriptive statistics, T-tests, and Chi-square tests assessed associations.

**Figure:**
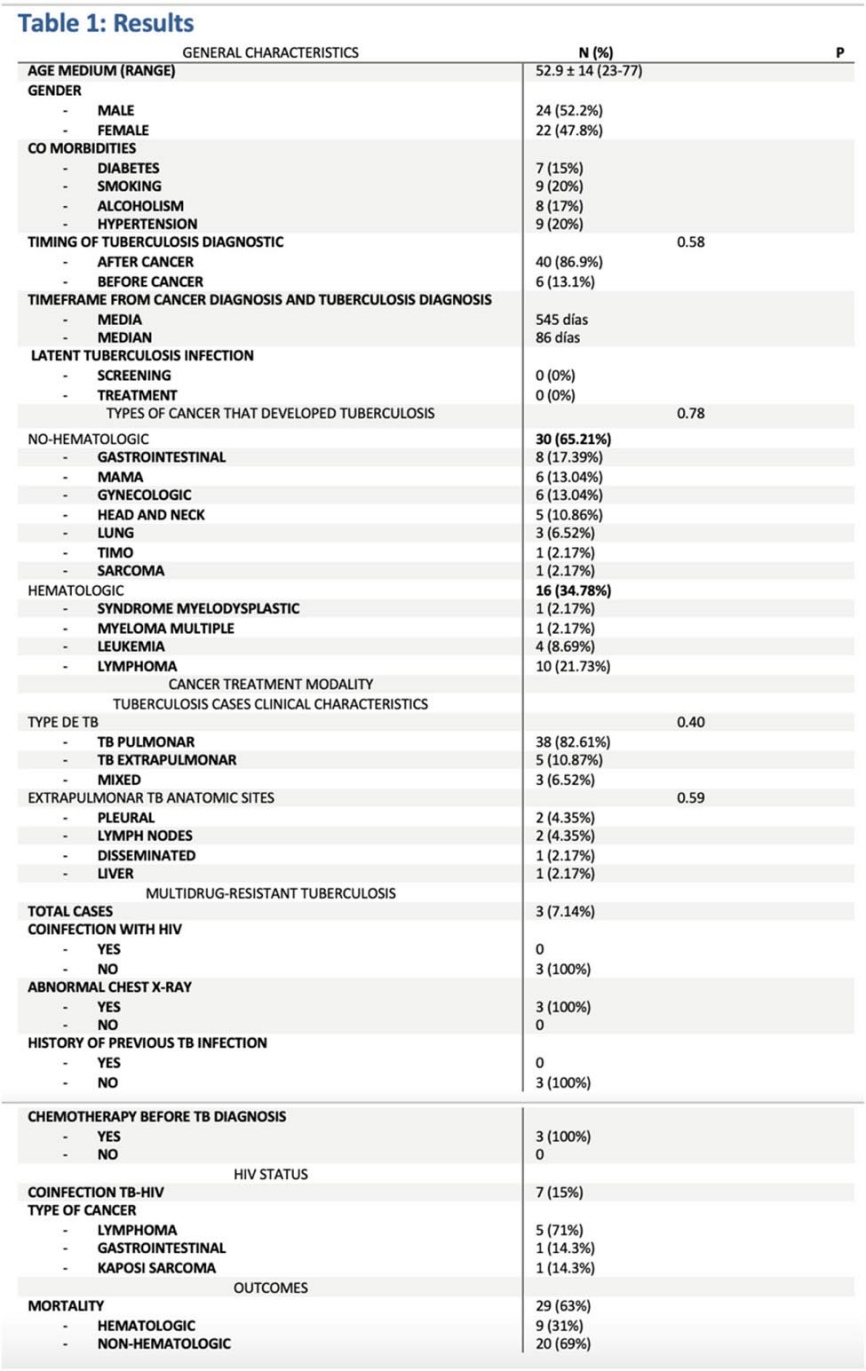
Results of the Retrospective Study of Tuberculosis cases reported in Patients with Cancer at the ION from 2012 to 2022.

**Figure:**
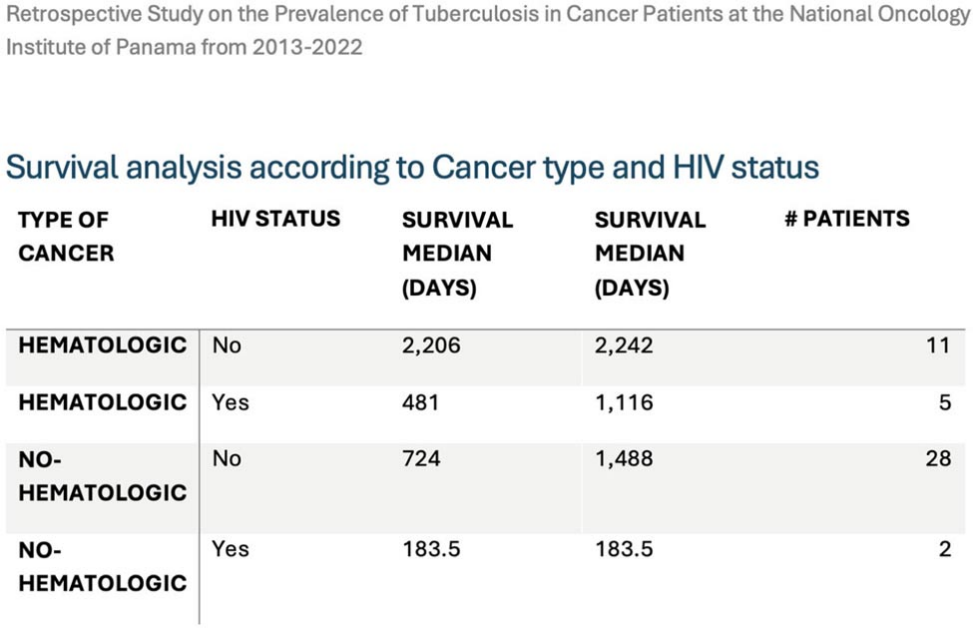

**Results:**

Among 70 TB cases at ION, 46 met inclusion criteria.

- Cancer Types: Non-hematologic (65.2%) included gastrointestinal (17.4%), breast (13.0%), gynecological (13.0%), head & neck (10.9%), and lung (6.5%). Lymphoma (21.7%) was the most common hematologic malignancy.
- TB Forms: Pulmonary TB was most frequent (82.6%), followed by extrapulmonary (10.9%) and mixed (6.5%).
- Drug-Resistant TB & HIV: Three MDR-TB cases (7.1%) had no HIV history, all post-chemotherapy. Seven (15%) had concurrent TB-HIV, mostly lymphoma cases. HIV-positive patients had lower median survival (337 vs. 871 days, p=0.174).

**Conclusion:**

Panama lacks structured TB screening for oncology patients. Hematologic cancers were linked to TB even without traditional risks. Immunosuppression (chemotherapy, HIV, comorbidities) likely contributed to TB reactivation. Three MDR-TB cases suggest possible undetected transmission. Limitations include ION’s lack of TB case management and small sample size. Future research should focus on early TB diagnosis and preventive strategies.

**Disclosures:**

All Authors: No reported disclosures

